# Abnormalities in male gametophytes development responsible for low seed set of *Peudosasa subsolida*

**DOI:** 10.1371/journal.pone.0316083

**Published:** 2025-01-30

**Authors:** Bonan Jiang, Zhihua Cao, Dejia Yang, Yongmei Wang, Yingchun Ma, Shiqi Zhang, Hui Zhan, Lixia Yu, Shuguang Wang, Juan Li

**Affiliations:** 1 The Key Laboratory of Forest Resources Conservation and Utilization in the Southwest Mountains of China Ministry of Education, Southwest Forestry University, Yunnan, China; 2 Provincial Key Laboratory for Conservation and Utilization of In-forest Resource, Key Laboratory of National Forestry and Grassland Administration on Biodiversity Conservation in Southwest China, College of Biological Science and Food Engineering, Southwest Forestry University, Kunming, China; 3 Anhui Academy of Forestry Sciences, Hefei, Anhui, China; 4 Shahe Forest Seed Breeding Center of National Long term Research Base, Chuzhou, Anhui, China; 5 Key Laboratory for Sympodial Bamboo Research, Faculty of Life Sciences, Southwest Forestry University, Kunming, Yunnan, China; 6 Institute of Bamboo and Rattan Science, Southwest Forestry University, Kunming, Yunnan, China; National Institute of Agricultural Research - INRA, MOROCCO

## Abstract

*Pseudosasa subsolida* belongs to the Pseudosasa genus within the Poaceae family. Due to its unique flowering cycle and the physiological traits associated with asexual reproduction, acquiring floral material from *P*. *subsolida* is particularly challenging. To investigate the causes of the low seed set rate in P. subsolida, floral organs and the development of male and female gametes were examined using conventional paraffin sectioning. The results revealed that the spikelet of *P*. *subsolida* exhibited the characteristics of a pseudospikelet with a latent bud, while the inflorescence displayed traits of an infinite inflorescence. Each spikelet contained approximately 10–16 florets and was accompanied by two bracts at its base. The fundamental structure of the florets comprised one lemma, one palea, three lodicules, three stamens, and one pistil. At the later stages of anther development, some abnormalities were observed, including the failure of pollen grains to form, deformation and shrinkage of the cells in the anther sac and tapetum, loss of the cells in the middle layer, cavitation of the microspores, and no identifiable contents The study concluded that the primary factor contributing to the low seed setting rate of *P*. *subsolida* was the aberrant development of male gametophytes. The significance of this study lay in its pioneering exploration of the reproductive structure of *P*. *subsolida*, and provide a theoretical reference for the fundamental examination of flower structure.

## Introduction

Bamboo, belonging to the Bambusoideae subfamily of the Gramineae family, represents one of the most significant forest resources, along with timber [1]. China possesses abundant bamboo resources, encompassing approximately 43 genera and 800 species. Bamboo forests cover approximately one-third of its overall land area [2]. The flowering cycle of bamboo plants is generally prolonged. Short-cycle bamboo species, such as *Melocanna baccifera* [3], typically take 10 to 15 years to flower. In contrast, the Shimen Moso Bamboo Forest in Fenghua, Zhejiang, is characterized by an exceptionally long flowering period, with no documented instances of flowering occurring for over 200 years [4]. The majority of bamboo species exhibit a low seed-setting rate or even the complete absence of fruiting [5]. Collecting flower and seed materials from fully developed bamboo plants therefore poses challenges that hinder research into their embryology. Currently, only a limited number of studies have provided detailed descriptions of the flower morphology and structure, as well as of megaspore occurrence and male/female gametophyte formation in bamboo species including *Bambusa multiplex* [6], *Bambusa rigida* [7], *Bambusa eutuldoides* [8], *Dendrocalamus sinicus* [9], *Fargesia yuanjiangensis* [10], *Neomicrocalamus prainii* [11], *Shibataea chinensis* [12], *Tongpeia fungosa* [13], etc.

*Pseudosasa subsolida* belongs to the Poaceae *Pseudosasa* and is characterized by its shrublike or small treelike growth and its rapid development. Additionally, it possesses significant ornamental value. The occurrence of this species is restricted to the Daliyu Mountain in Yiyang County, Hunan, China (112.327023*°*N,28.590358*°E*). Being a natural wild species, it thrives exclusively in hilly terrains and yellow soil [14, 15]. About the morphological characteristics of its adult bamboo, slight grooves can be observed at the base of branching internodes, where the rod wall is thick and nearly solid, with a medullary spongy structure. These characteristics contribute to exceptional firmness, resilience, and durability. *P*. *subsolida* is the preferred material for furniture and carving handicrafts [16].

Bamboo species infrequently undergo flowering, and their floral structure generally aligns with that of other plants in the Gramineae family. However, certain bamboo flowers exhibit a regression into scales, while other components regress into membranes, resulting in relatively diminutive sizes. The bamboo flowers were distinguished by their absence of vibrant hues and fragrant aromas, as well as their unique shapes. Angio-sperms utilize flowers as the fundamental structural unit for the formation of inflorescences, whereas bamboo inflorescences exhibit a higher level of complexity, being composed of spikelets. Thus, spikelets are the constituents of bamboo inflorescences [17].

Currently, the research on *P*. *subsolida* primarily focuses on species classification and morphological description, leaving significant gaps in other areas of study. There is a lack of records regarding flowering and other aspects of *P*. *subsolida* research. Examining its microstructure using the paraffin section method is even more limited. Because of the characteristics of bamboo plants, such as difficulty in flowering, and only flowering but no fruity, there are great obstacles in the breeding research of *P*. *subsolida*. For its special physiological characteristics, the reasons for the abortion of the floreus of its species should be first revealed. Then further solutions should be found, to provide effective help for breeding. It is known from the Flora of China [18] that *P*. *subsolida* has been evaluated as a vulnerable species, and the change in its ecological environment has led to the continuous decline of its species population. Therefore, it is of great significance to study the reproductive organ florets of *P*. *subsolida* and find out the reasons for their low seed setting rate to improve their physiological characteristics of asexual reproduction and protect endangered species The morphological structure of the floral organs of *P*. *subsolida* is described in this study, aiming to complement the known classification characteristics of *P*. *subsolida*. Additionally, the development of male and female gametophytes, as well as the changes in the anther wall structure, is investigated in *P*. *subsolida* flowers to further elucidate the factors contributing to its low seed-setting rate. This study provides original embryological data for future research on *P*. *subsolida* and establishes a foundation for breeding efforts.

## Material and methods

**Fig 1:**
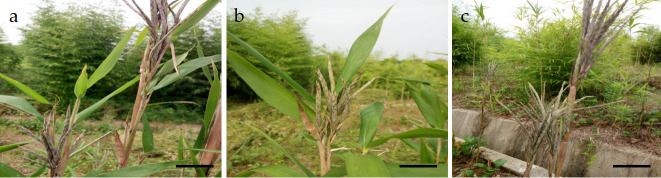
Floater morphology of *P*. *subsolida* in the wild. (a, b, c) Spikelet morphology. Note: The scale bars are uniformly 1 cm.

In 2018, spikes from six bamboo plants at the flowering stage were collected, and a total of 60 spikes were selected after pooling them. at the Shahe Forest Seed Breeding Center of National Long term Research Base, Anhui Province (31.851156*°*N,117.181445*°*E) (Fig 1a–1c). A comprehensive anatomical study on the floral organs and the development of female and male gametes of *P*. *subsolida* was conducted in 2021 at Southwest Forestry University (Kunming,Yunnan,China) to investigate the influencing factors of its low seed setting rate, utilizing routine paraffin section methods. In the selection of spikelets, only those that were healthy, free from disease or damage, and at an appropriate developmental stage (usually flowering) were considered. Additionally, the selected spikelets were representative of the population and free from hybridization influences to ensure genetic purity. The spikelets were placed in a 50% FAA stationary solution (50% ethanol: 40% formaldehyde: glacial acetic acid = 18: 1: 1) and the sample bottle containing the spikelets was opened and placed under a vacuum pump for 8 hours to remove air from the sample and expedite its fixation. The FAA-fixed spikelets were transported to the laboratory under low-temperature conditions, and a total of 15 spikelets exhibiting good developmental status were carefully selected for subsequent anatomical experiments.

The *P*. *subsolida* spikelets were taken from the FAA fixation solution and dissected under an anatomical microscope (Olympus H011). Before dissection, the spikelet, floret, and spikelet axis were photographed and their lengths measured; then, the lemma, palea, pulp, pistil, and stamen of the spikelet were dissected, photographed, and measured, and the data were statistically processed using Excel software. The anatomized stamens and pistils were dissected according to Li Zhengli’s paraffin continuous section method [19]. After dissection, the florets, anthers, and ovaries were dehydrated, immersed in paraffin, and cut into 7μm thick sections using a Leica RM2165 microtome. After that, the slices were stained twice, with 1% saffron and 1% solid green, and dehydrated in 50% xylene and 100% xylene. The slice images were selected under a Nikon-ECLIPSE 50 microscope and measured with DS-3000 two-dimensional measurement software. To ensure accuracy, each microscopic morphological feature was observed and measured three times, and a total of 60 slides were prepared to minimize the potential errors in microscopic observation [20].

Ethics approval and consent to participate:

Not applicable. The authors declared that experimental research works on the plants described in this paper comply with institutional, national and international guidelines. Use of plant material has been permitted.

## Results and discussion

### Flower morphology and anatomy of *P*. *subsolida*

**Fig 2:**
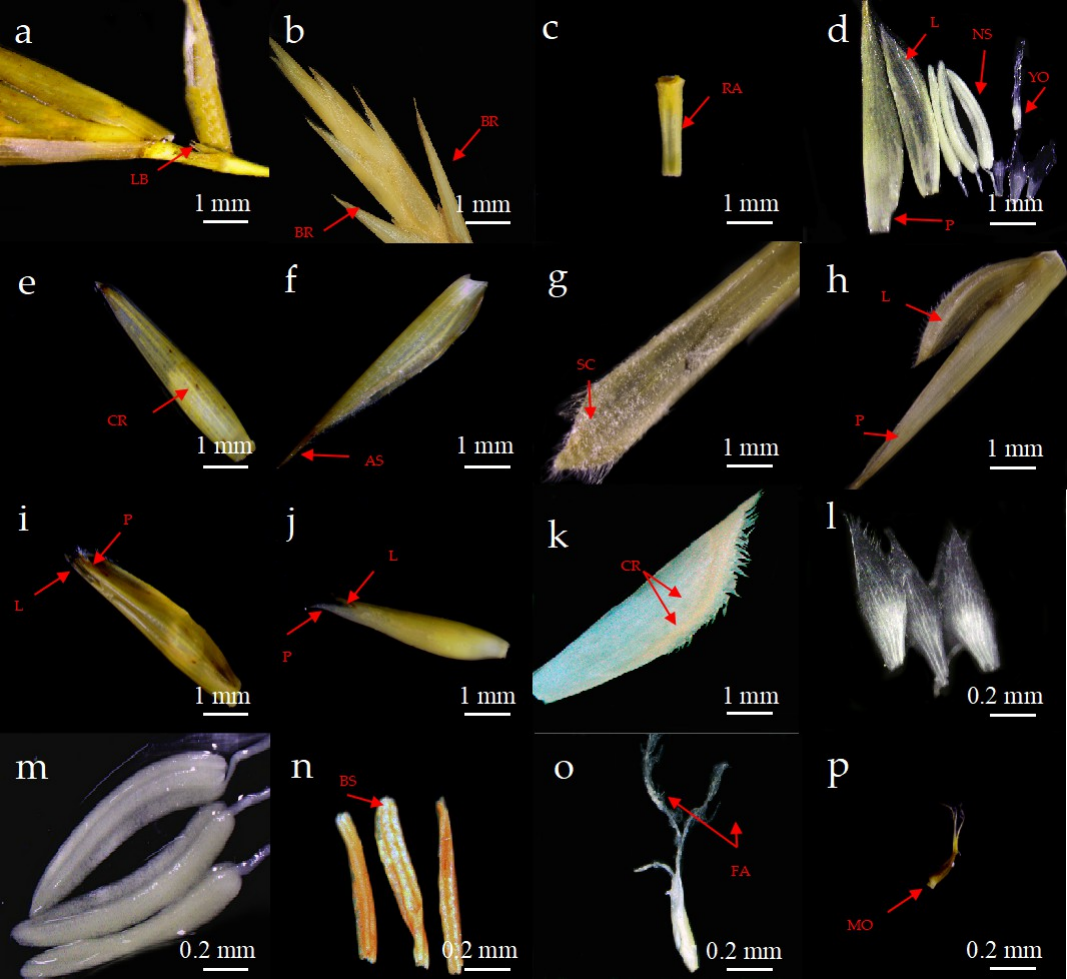
The morphological anatomy of *P*. *subsolida*. (a) LB: Latent bud. (b) BR: Bracts. (c) RA: Rachilla. (d) Whole floret. L: Lemma. P: Palea. NS: Normal stamens. BS: Brown stamens. YO: Young ovary. (e) Lemma, CR: coarsest ridge. (f) AS: apex sharp. (g) SC: surface ciliated. (h) Lemma was longer than the palea. (i) Lemma was equal in length to the palea. (j) Palea was slightly longer than the lemma. PA: palea. LE: lemma. (k) Palea had two ridges, ciliated; CR: coarsest ridge. (l) lodicules. (m) Normal stamens. (n) Brown stamens. (o) Young ovary, FA: feathery appendages. (p) MO: Mature ovary.

The spikelet base of *P*. *subsolida* exhibited latent buds (Fig 2a), also known as pseudo spikelets(Note: The spikelets of bamboo plants are classified into true and false spikelets based on the presence or absence of latent buds in the bracts at the base or in the axils of the glumes. True spikelets lack latent buds, whereas false spikelets contain latent buds or exhibit emerging leaves.). The inflorescence of *P*. *subsolida* consists of pseudo-spikelet growth on various levels of vegetative branches, called indefinite inflorescences [21]. Anatomical observations of the flowers of *P*. *subsolida* revealed that two bracts were present in close proximity to the floret at the base of the spikelet (Fig 2b). The average length of twenty randomly selected spikelets was 6.42 cm, with each spikelet containing 10–16 florets. The uppermost floret among these exhibited sterility, characterized by a young ovary devoid of stamens. Subsequently, a total of 50 florets were randomly selected, and their average length was determined to be 1.36 cm. Adjacent florets within the same spikelet were interconnected through the rachilla (Fig 2c), exhibiting alternate arrangement along the spikelet axis with cilia present. Furthermore, the mean length of 50 spikelet axes was measured to be 0.47 cm.

The anatomical examination of a complete floret revealed that it comprised (from outside to inside) one lemma, one palea, three lodicules, three stamens, and one pistil (Fig 2d). The average length of the lemma was 1.35 cm, and it had 9–12 longitudinal veins, with the thickest and longest vein positioned at the center (Fig 1e). Additionally, cilia were observed on the surface, with long cilia present at the edge of the apex, which featured a sharp tip (Fig 2f and 2g). The palea was found to be paired with the internal growth of the lemma. the length of the lemma in the mature florets of *P*. *subsolida* was slightly greater than that of the palea (Fig 2h), whereas in immature florets, there was minimal difference in length between them (Fig 2i), except for a few instances where the palea extended beyond the lemma (Fig 2j). The palea exhibited two prominent ridges accompanied by four veins positioned between the ridges and three to four veins on each side. Notably, the surface of the palea was adorned with cilia, which were particularly elongated at the apex edge (Fig 2k). Three bracts (Fig 2l) surrounded the ovary; two were located near the lemma and one was covered by the palea. These bracts were thin and possessed white transparent membranous structures. During flowering, the bracts absorbed water and expanded, causing the lemma to spread open. The floret comprised three stamens (Fig 2m). The stamens were typically light yellow but showed browning in some instances (Fig 2n).

The surface of the ovary exhibited a smooth and glabrous texture, appearing white in its juvenile stage (Fig 2o). Subsequently, as it reached maturity, the ovary underwent a gradual browning process (Fig 2p). The style was characterized by its short length, while the stigma displayed a trilobed structure with feathery appendages.

### Microsporogenesis and male gamete development of *P*. *subsolida*

#### Microsporogenesis

**Fig 3:**
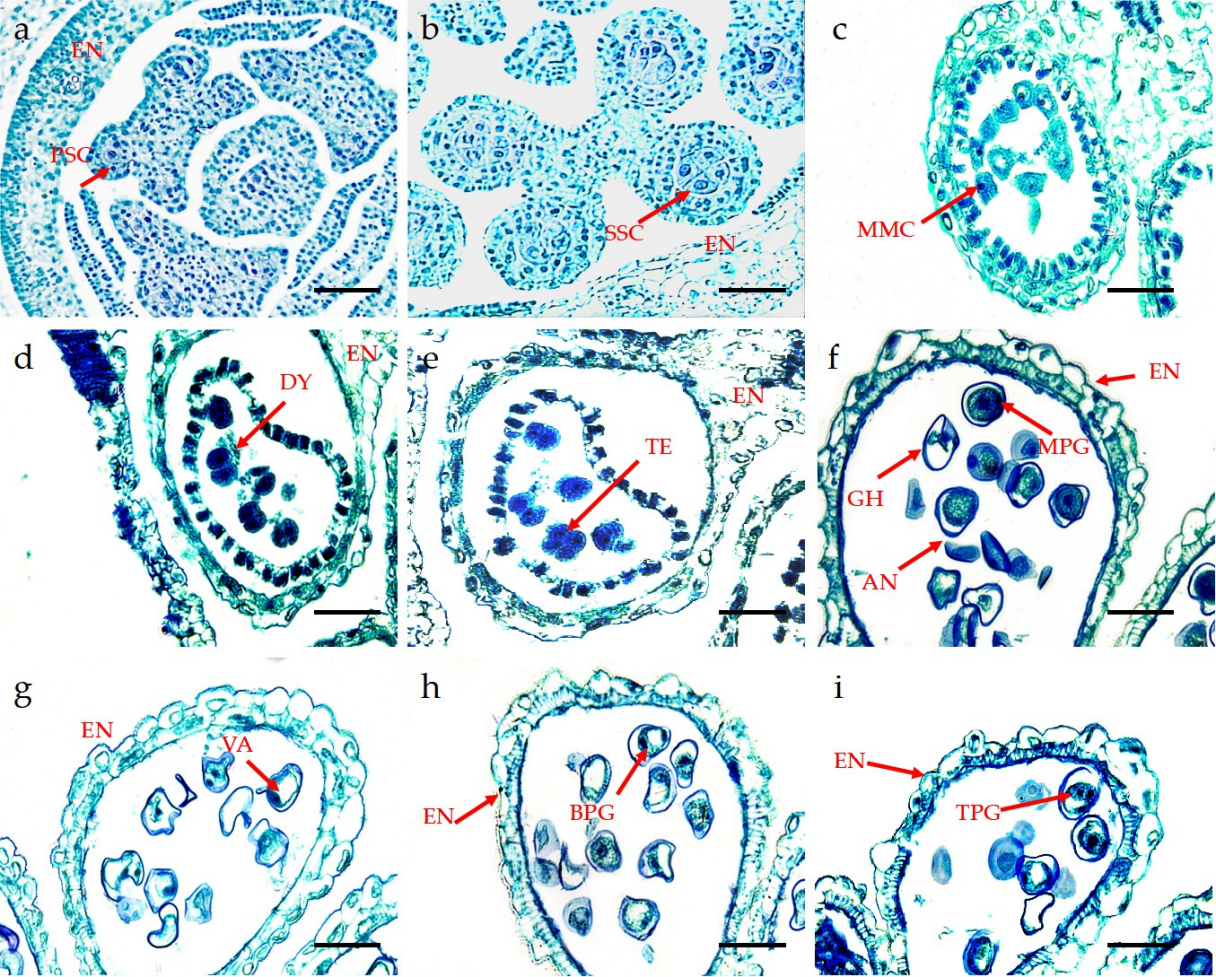
The anatomical structure of the anther of *P*. *subsolida*. (a) PSC: Primary sporogenous cells. (b) SSC: Secondary sporogenous cells; (c) MMC: Microspore mother cell; (d) Dichotomous period, DY: dyad; (e) TE: Tetrad period; (f) MPG: Mature pollen grains, GH: germination holes; (g) Large vacuoles in the center of cells; VA: vacuoles; (h) BPG: Binucleate pollen grains; (i) TPG: Tri-nuclear pollen grains. Note: The scale in the figure is 50μm.

*P*. *subsolida* possesses three stamens, each stamen has an anther with four chambers that contain the sporogenous tissue. During the early stages of anther development, the outer epidermis comprises a layer of flattened cells, while inside the epidermis lies a cluster of cells with similar morphological structures. Notably, the cells located in the four locules of the anther divide more rapidly than other cells, leading to the gradual differentiation of sporogenous cells beneath each corner’s epidermis. Subsequently, these sporogenous cells initiated periclinal divisions, dividing outward to form the primary parietal layer and inward to generate primary sporogenous cells (Fig 3a). The primary sporogenous cells continue dividing and differentiate into secondary sporogenous cells (Fig 3b), which subsequently differentiate into larger microspore mother cells devoid of distinct vacuoles (Fig 3c). These microspore mother cells undergo subsequent developmental stages involving meiosis. At the conclusion of the first meiotic division, a dyad was formed by the microspore mother cell (Fig 3d), and a symmetrical tetrad was subsequently generated after meiosis II (Fig 3e). Consequently, the cytoplasmic division mode exhibited by the microspore mother cell adheres to a continuous type.

### Development of male gametes

The anthers progressed through the tetrad stage before transitioning into the microspore stage. Upon the dissolution of callose, the four daughter cells within the tetrad underwent separation and gave rise to autonomous mononuclear pollen grains, known as microspores. Immediately following release from the tetrad stage, the microspore exhibited a dense cytoplasm, with the nucleus occupying the central region of the cell, while vacuole formation had not yet occurred. This developmental phase was referred to as the systolic or early microspore phase (Fig 3f). As the volume of the microspores increased, vacuoles appeared in the cytoplasm, and the nucleus began to migrate towards one side of the cell. Simultaneously, gradual cell wall formation took place until complete nuclear relocation and extensive vacuolation in the middle region of the cell were achieved. The appearance of the germination hole (Fig 3f) was referred to as the mononuclear margination stage (Fig 3g). Subsequently, gradual cytoplasmic filling occurred throughout the entire cell. Concurrently, the nuclear division took place through mitosis, resulting in the formation of a binucleate pollen grain where one nucleus was divided into two nuclei (Fig 3h). The division resulted in two distinct cells: one, located adjacent to the pollen wall, functioned as a reproductive cell; the other, situated in the central part of the cell, served as a nutritive cell. In mature pollen grains observed from *P*. *subsolida* florets, predominantly binucleate pollen grains were found, with a small proportion consisting of trinucleate ones (Fig 3i).

#### Development of the anther wall

**Fig 4:**
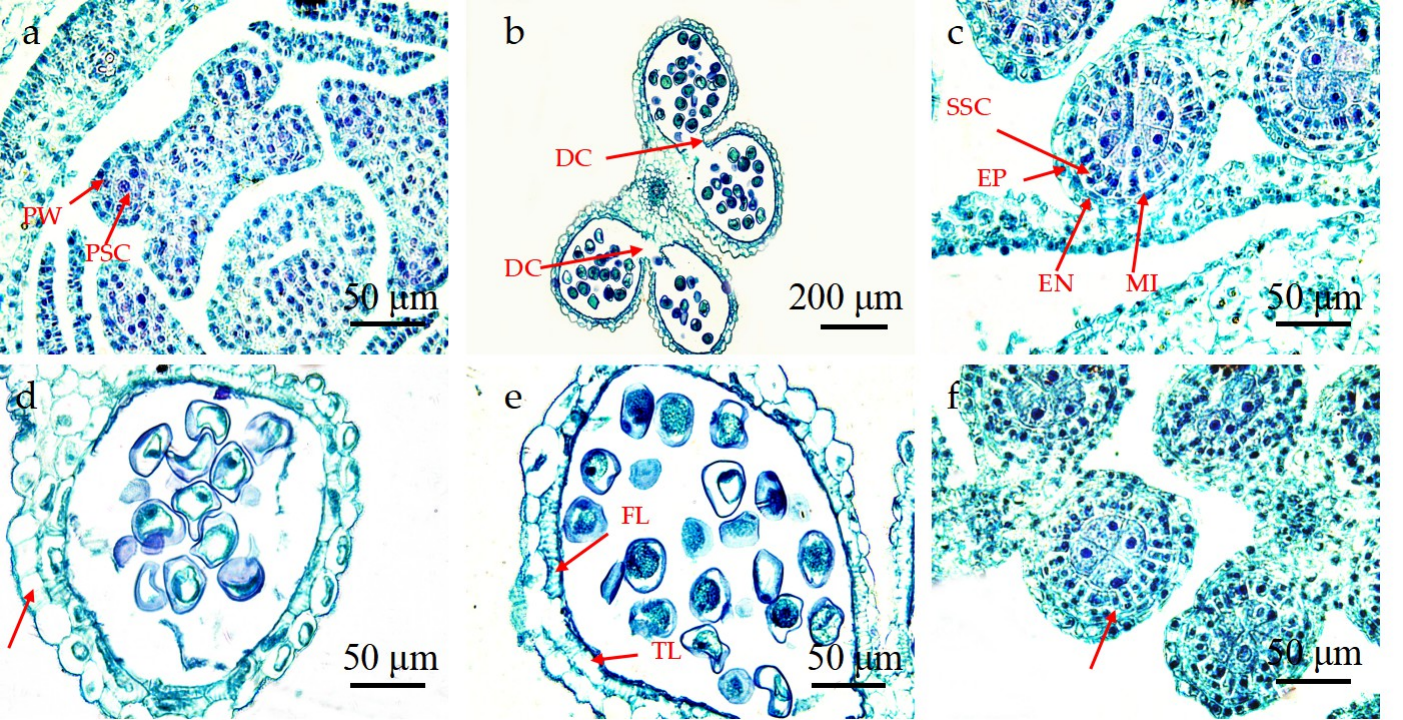
Anther wall development and abortive anthers of *P*. *subsolida*. (a) Primary wall and primary sporulation cells. (b) The septum of adjacent drug chambers disappeared, and the drug chambers communicated. (c) The secondary sporulation stage had four layers of walls; the epidermis cells were shown in the sporulation stage. (d) Microspore stage. (e) Tapetum degradation and the disappearance of the middle layer. (f) Secondary sporulation period. PW: Primary wall. PSC: primary sporulation cells; DC: drug chambers; EN: endothecium; EP: epidermis; MI: middle layer; SSC: secondary sporogenous cell; TL: Thin layer, FL: Fiber layer.

The young stamens consisted of an epidermal layer and sporogenous cells when observed from a transverse perspective, while the epidermal cells maintained a consistent monolayer throughout the entire developmental process. The formation of primary parietal cells and primary sporogenous cells occurred both inwardly and outwardly during peripheral division (Fig 4a). The inner layer of the primary wall differentiated into the middle layer and tapetum, while the outer layer gave rise to the endothecium. Through secondary spore production, all cell layers including the epidermis, endothecium, middle layer, and tapetum underwent differentiation during anther development (Fig 4b). In the later stages of pollen maturation, a small aperture formed between two adjacent locules to connect the two chambers (Fig 4c), resulting in the formation of four longitudinal chambers for the efficient release of pollen grains. The epidermis persisted throughout development and underwent periclinal division to accommodate the expansion of internal tissues within the locule walls while providing them with protection. During the spore production phase, the epidermis assumed a rectangular shape with a discernible nucleus (Fig 4d). However, during microspore development, its morphology became irregular, accompanied by the near disappearance of the nucleus and the formation of a cuticle layer (Fig 4e). The inner wall of the locules underwent radial elongation through striplike additions on its internal surface. As the anthers matured, their fibers progressively thickened as they underwent radial elongation, leading to longitudinal splitting in this region to facilitate the release of pollen grains (Fig 4d).

During another developmental stage, the middle layer existed transiently, with its constituent cells assuming a rectangular morphology during the secondary sporogenesis phase (Fig 4b). During microspore meiosis, the middle layer underwent significant reduction and eventually disappeared completely upon anther maturation. The development of the tapetum was intricately linked to microspore formation and male gametophyte development. During the microspore stage, the tapetum played a crucial role in providing essential nutrients for micro-spore development. During the secondary sporulation stage, there was a noticeable increase in density within the tapetum cytoplasm (Fig 4f). Afterward, the microspore initiated disintegration during the later stage and ultimately underwent complete disintegration upon pollen maturation, leaving behind only residual thin layers in their original position. Consequently, the tapetum layer of *P*. *subsolida* anthers should classified as the glandular type.

### Anther abortion types of *P*. *subsolida* florets

**Fig 5:**
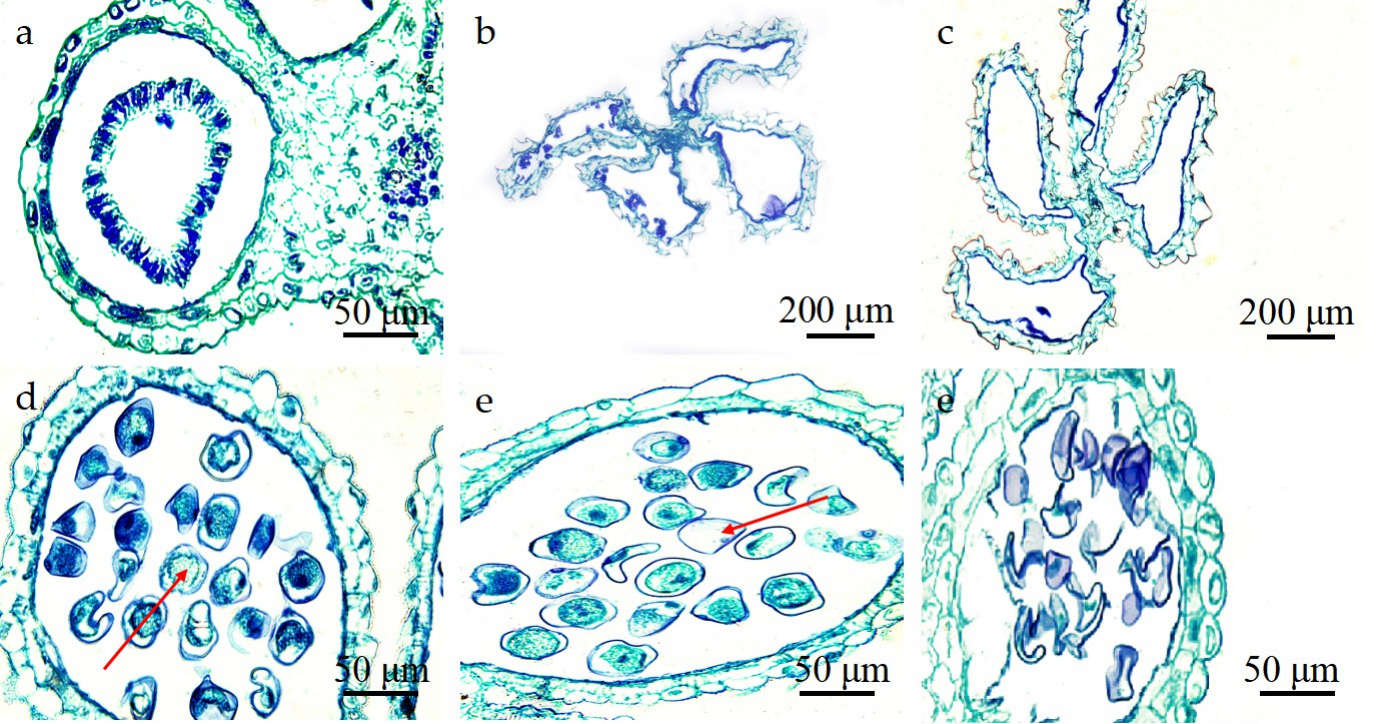
Anther abortion type of *P*. *subsolida*. (a) The capsule was hollow and no pollen grains formed; (b) Shrinkage deformation of pollen grains; (c) The tapetum and middle layer cells degenerated and no microspore formed; (d) Some microspores had no nucleus; (e) Pollen grains had no nucleus or cytoplasm; (f) Contracted and abortive pollen grains.

Although *P*. *subsolida* underwent flowering, its fruiting rate was extremely low, resulting in a scarcity of harvestable seeds. Sliced observations of the anthers of *P*. *subsolida* revealed a phenomenon of sterile anthers. These sterile anthers were classified into 6 types based on our analysis: complete absence of pollen grains throughout the locule (Fig 5a); Locules contraction and deformation squeeze the internal space, subsequently affecting pollen grain development. (Fig 5b); failure to form a tapetum layer and middle layer cells (Fig 5c); the presence of empty microspores lacking cell nuclei or undergoing incomplete vacuolation or contraction phase (Fig 5d); normal pollen grains but some without a cell nucleus and cytoplasm to provide nutrients for normal pollen grains (Fig 5e); The sterile pollen grain eventually shrinked and deformed, and the pollen grain wall was inwardly depressed to form a crescent contraction state (Fig 5f).

### Megasporogenesis and anatomical structure of the ovary

**Fig 6:**
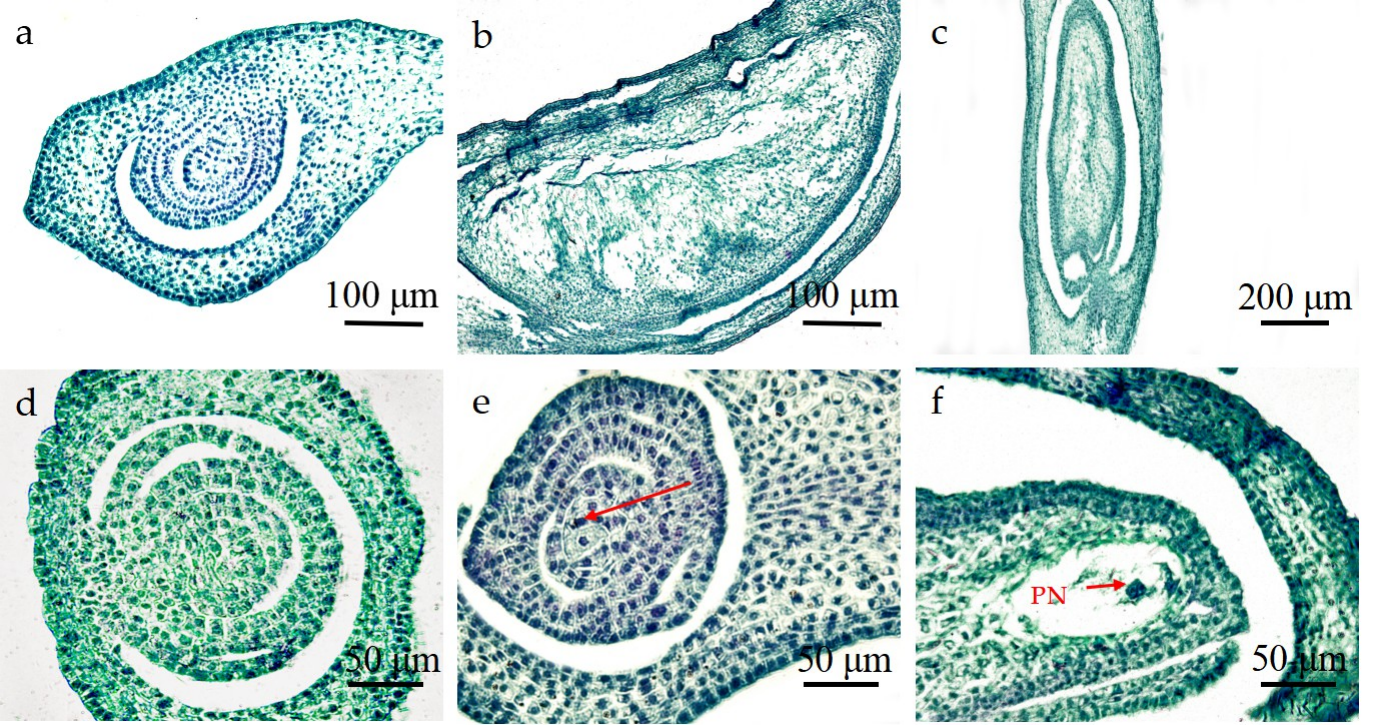
Megaspore and female gametophyte of *P*. *subsolida*. (a, b) Anatropous ovule, M: micropyles; (c, d) double-layer integument; (e) Megasporocyte; (f) Central cell. PN: Polar nucleus.

The ovules of the ovary of *P*. *subsolida* had their micropyles (The micropyle is a small opening or gap at the top of the ovule in seed plants, formed due to the nonunion of the integuments.) facing inward, toward the center of the ovary (Fig 6a and 6b), and were surrounded by two layers of integument (Fig 6c and 6d). When the sporogenous cells initiated differentiation, an outer integument was formed surrounding the inner integument, while the archesporial cells continued to grow and directly developed into megasporocytes (Fig 6e). Subsequently, each megasporocyte underwent two rounds of meiosis, resulting in the formation of a dyad and a tetrad, respectively. With each tetrad, one functional megaspore developed adjacent to the micropyle, while the other three degenerated. The functional megaspore underwent multiple mitotic divisions until it reached maturity as an embryo sac. During embryo sac maturation, there was a central cell composed of two parallel polar nuclei positioned between the egg apparatus and antipodal cells (Fig 6f). This central cell served as a precursor to the endosperm and represented the largest cell within the embryo sac.

## Discussion

### Comparison of the morphological structure of floral organs

Bamboo species exhibit significant variation in terms of the size and shape of their bamboo florets, as well as the spikelets, cobs, stamens, pistils, and bracts of the inflorescence structure. There were disparities in the morphology and length of flower branches between bamboo species. The flower branches of *P*. *subsolida* are densely arranged, with clustered spikelets distributed flatly, resembling the morphological characteristics observed in *Bambusa oldhami* [22] and *D*. *sinicus* [9]. The morphological features of *Chimonobambusa utilis* [23] and *Sasaela kongosanensis* [24] were different.

To further investigate the specific structure of florets, an examination of the morphological anatomy of floret organs revealed that the majority of bamboo species have bisexual florets. *P*. *subsolida*, similar to *Phyllostachys praecox*, [25], *Chimonobambusa utilis* [23], and *Shibataea chinensis* [12], possesses three stamens in its floret organ. The number of stamens in *Dendrocalamus hamiltonii* [20], *Bambusa intermedia* [26], *B*. *multiplex* [6], and *D*. *sinicus* [9] was six. *Pistils of P*. *subsolida* similar to the conspecific *P*. *viridula* [27], which exhibits densely ciliated and nondesquamated apices, in contrast to *D*. *hamiltonii* [20]. The six-stamen bamboo species included *Dendrocalamus* and *Bambusa*. The styles of *P*. *subsolida* are short, as indicated by the length of its stigma [28]. In bamboo plants, florets can be divided into long and short styles; therefore, *P*. *subsolida* was indicated rather than the congeneric *P*. *viridula* type because of the short styles. The mature lemma of the floret was slightly longer than the palea, while the length of the immature lemma did not differ significantly. The lemma exhibits a sharp tip and ciliated edges, with the palea positioned internally. These characteristics were similar to those of the conspecific *P*. *viridula* [27]. In *P*. *subsolida*, *Bambusa oldhami*, *Bambusa multiplex*, *Bambusa intermedia*, *Bambusa rigida*, *Shibataea chinensis*, *Pseudosasa viridula*, and *Chimonobambusa utilis*, the shape of the pistil was mostly a three-branched stigma; in *Dendrocalamus hamiltonii* and *Dendrocalamus sinicus*, there was an unbranched pistil, while the stigma of *Arundinaria simonii* contains two branches.

Bamboo plants are distinguished by their towering stature and predominantly wind mediated dispersal. In terms of pollination mechanisms, it is more difficult for wind dispersed plants to achieve widespread distribution compared to insect pollinated counterparts. Therefore, bamboo plants exhibit sporadic flowering patterns. Flowering and subsequent seed production in bamboo are rare, with limited or nonexistent seed collection even during sporadic bamboo flowering. As for the flower type, *P*. *subsolida* has continuous bloomsplants that do not decline after flowering but instead undergo asexual reproduction to renew the bamboo forest. The low seed-setting rate can be attributed to the varying types and periods of flowering observed in bamboo plants, along with potential insect infestation (in studies of *P*. *viridula*) [27].

Combined with bamboo habitat, this study provided a more nuanced description of bamboo reproductive biology. In the field of bamboo plant reproductive biology, McClure [29] initially proposed the concept of “pseudo” spikelets and subsequently classified bamboo plant inflorescences into two categories based on the presence or absence of dormant buds located at the base of spikelets: determinate inflorescence and indeterminate inflorescence. In 1986, Geng Bojie provided a more precise definition of bamboo inflorescence as a finite true inflorescence occurring only once and an infinite false inflorescence with successive occurrences [30]. According to Zhang Zuxin’s research literature, employing gene editing technology to knock out genes in the inflorescence can significantly reduce the abortion rate of maize florets, suggesting a potential association between inflorescence and this phenomenon [31]. No existing literature has been found regarding the correlation between the structure of false spikelets and inflorescences and abortion.

Florets of bamboo plants are contained in false spikelets, and the flowering process of florets is described. According to the flowering dynamics of florets in bamboo plants, upon blooming, water absorption by the pulp leads to lemma expansion. This facilitates the emergence of both male and female stamens from the lemma sheet while ensuring their simultaneous maturation. Upon completion of the powdering process, the pulp undergoes desiccation and contraction, resulting in the closure of the lemma sheet and retraction or abscission of stamens. This floral morphology corresponds to an open type(Open type are particularly common in Bambusa. During flowering, the cells absorb water, causing the lemma segments to expand, and the filaments to elongate.). When in bloom, the lemma remains closed, with simultaneous maturation of both the pistil and stamen. This phenomenon can be classified as a closed type [17]. The most apparent distinction between the two types lies in the presence of a pulp sheet in the open type, whereas the closed type lacks such a component. In this study, *P*. *subsolida* was found to have pulp flakes and to be an open floret. In this respect, its floral features were very similar to those of Bambusa. By contrast, *Dendrocalamus sinicus* and *D*. *hamiltonii* did not have pulp and are of the closed type(closed-type is exemplified by Dendrocalamus, whose florets lack pulp and whose lemma does not open during flowering.).

In this study, *P*. *subsolida* was found to exhibit infrequent blooming and a significantly low seed-setting rate within its native habitat, with no naturally occurring seedlings observed. The cytokinesis mode of the pollen mother cell was classified as the continuous type. This type was completely consistent with the spore development of *M*. *sichuanensi* [32] and was the same as that of *D*. *sinicus* [9]. However, most of the microspore tetraploids produced by *D*. *sinicus* are tetrahedral. The resulting dizygomorphic tetraploid was similar to that of *B*. *multiplex* [6], although the process of formation was not the same. The cytokinesis type of *B*. *multiplex* was simultaneous, resulting in the absence of diploid formation at the end of the first division of meiosis and direct tetraploid formation during the second division. During secondary sporulation, anther wall development involves four layers of cells: the epidermis, anther chamber wall, mesosphere, and tapetum. The anther walls of *B*. *eutuldoides* [8] and *B*. *intermedia* [26] were also fully differentiated during the secondary sporulation period, which was consistent with the development of the anther wall of *P*. *subsolida*. In contrast, the anther walls of *D*. *sinicus*, *M*. *sichuanensi*, and *B*. *multiplex* [6, 9, 33] were fully differentiated at the microspore mother cell stage. The anther wall of *P*. *praecox* differs from that of *P*. *subsolida* in that it lacks an inner layer, which typically consists of (from outer to inner) the epidermis, middle layer, and tapetum. The majority of mature pollen grains in *P*. *subsolida* exhibited binucleate characteristics, while a minority displayed trinucleate features. The pollen of *M*. *sichuanensi* [32] and *S*. *chinensis* [12] was similar to that of *P*. *subsolida*.

### Reasons for the low seed-setting rate

The potential causes of spontaneous abortion in *P*. *subsolida* included the presence of an in-distinct or absent demarcation between tapetal cells and intermediate cells. The primary role of the tapetum was to provide nourishment and structural components for microspores [34]. During the later stages of anther development, the tape-tum undergoes deformation and releases lipids or phenols that were essential for proper anther development [35]. During the pollen grain development of *P*. *subsolida*, similar to that in most bamboo species (e.g., *B*. *multiplex*, *B*. *sinospinosa*, *Shibataea chinensis* [6, 26, 36], there was abnormal tapetum development. In contrast to *D*. *sinicus* [26], in which the tapetum is normally developed, in *D*. *sinicus*, the tapetum begins to disgroup at the stage of microspore mother cells, but it remains in its original position and does not disappear. However, in *P*. *subsolida*, although the tapetum and midlayer persist during the microspore mother cell stage, they completely disappear during meiosis and cannot provide the necessary nutrients for normal microspore development. *Phyllostachys edulis* [37, 38] has also been reported to have flowering (flowering in all seasons, all developmental stages) and normal fruit-bearing. Still, the anatomical structure of the female and male gametes in *Phyllostachys edulis* had not been described. The second point pertains to the presence of hollow microspores, which do not possess characteristics typical of pollen grains. In this study, anther shrinkage was observed during the development of *P*. *subsolida* florets; a similar phenomenon was observed in *Neomi-crocalmus praini* and *B*. *tuldoides*. Additionally, *B*. *intermedia* [11, 26, 36] exhibited the contraction of other compartments, as observed in *P*. *subsolida*. However, it is hypothesized that *S*. *chinensis* exhibits limited and potentially negligible seed setting due to factors such as concealed stigma hindering effective pollination, self-pollination, and challenges associated with dioecious maturation. No instances of abnormal structural development induced abortions were observed in female or male gametophytes of *D*. *sinicus* [9] through paraffin continuous section analysis. It was worth noting that potential factors contributing to the low seed-setting rates in *P*. *subsolida*, similar to those observed in the aforementioned bamboo species, cannot be completely disregarded. Thus, there was a need for further research.

### The application uses and characteristics for future research

Morphological examination and dissection of the female and male gametes of *P*. *subsolida* will help to fill the gaps in the understanding of the reproductive structure of this genus and its conspecifics. The anatomical techniques used in this study may provide valuable references for other anatomical studies of floret structures. In addition, the present study revealed the root cause of low or no seed setting rate in Bamboos pauciphylla, providing insights into the physiological characteristics of the asexually propagated bamboos pauciphylla species. In future studies, advancements in bamboo breeding techniques, coupled with the shift from asexual reproduction via cuttings and transplants to sexual reproduction through seed use, could further enhance our understanding of the causes of floret sterility in bamboos.

## Conclusions

Based on the anatomical study of the florets of *P*. *subsolida*, the results obtained were as follows: The inflorescence of the *P*. *subsolida* floret was classified as an indeterminate inflorescence with dormant buds located at the base of the spikelet, exhibiting a pseudo-spikelet morphology. The inflorescence consists of 10–16 florets accompanied by two bracts positioned at the base of the spikelet. Each floret comprises a lemma, a palea, three lodicules, three stamens, and one pistil. The stamens were within a four compartment longitudinal fissure, with the anther wall comprising four layers of cells: the epidermis, inner anther wall, middle layer, and tapetum. Among these layers, the tapetum was glandular in nature. The cytokinesis of the pollen mother cell was classified as continuous type. The ovary was one locule and superior, featuring a feathery three forked stigma, anatropous ovule, parietal placenta, and two integuments, with partial browning observed in certain fertile florets. The maturity of florets gradually decreases from the base to the top of the spikelets, where the uppermost floret exhibits infertility due to a young ovary and the absence of stamens. The majority of pollen grains were binuclear or trinuclear; however, abnormal development of their anthers and the occurrence of brown stamens can be observed in fertile florets. Anther abortion was the primary contributor to the low seed-setting rate observed in *P*. *subsolida*. This study makes a significant contribution to the morphological investigation of bamboo florets and establishes a solid foundation for the taxonomic classification of bamboo species. Its importance lies in the pioneering exploration of the reproductive structure of P. subsolida. However, a key limitation is the scarcity of studies on flower structures in this genus, which hinders meaningful comparisons and prevents the identification of more precise common features.
